# Basic Research Approaches to Evaluate Cardiac Arrhythmia in Heart Failure and Beyond

**DOI:** 10.3389/fphys.2022.806366

**Published:** 2022-02-07

**Authors:** Max J. Cumberland, Leto L. Riebel, Ashwin Roy, Christopher O’Shea, Andrew P. Holmes, Chris Denning, Paulus Kirchhof, Blanca Rodriguez, Katja Gehmlich

**Affiliations:** ^1^Institute of Cardiovascular Sciences, College of Medical and Dental Sciences, University of Birmingham, Birmingham, United Kingdom; ^2^Department of Computer Science, University of Oxford, Oxford, United Kingdom; ^3^Institute of Clinical Sciences, College of Medical and Dental Sciences, University of Birmingham, Birmingham, United Kingdom; ^4^Stem Cell Biology Unit, Biodiscovery Institute, British Heart Foundation Centre for Regenerative Medicine, University of Nottingham, Nottingham, United Kingdom; ^5^University Heart and Vascular Center, University Medical Center Hamburg-Eppendorf, Hamburg, Germany; ^6^Cardiovascular Medicine, Radcliffe Department of Medicine, University of Oxford and British Heart Foundation Centre of Research Excellence Oxford, Oxford, United Kingdom

**Keywords:** heart failure, *in vivo* cardiac models, human induced pluripotent stem cells, methods, *in silico* modelling, cardiac arrhythmias

## Abstract

Patients with heart failure often develop cardiac arrhythmias. The mechanisms and interrelations linking heart failure and arrhythmias are not fully understood. Historically, research into arrhythmias has been performed on affected individuals or *in vivo* (animal) models. The latter however is constrained by interspecies variation, demands to reduce animal experiments and cost. Recent developments in *in vitro* induced pluripotent stem cell technology and *in silico* modelling have expanded the number of models available for the evaluation of heart failure and arrhythmia. An agnostic approach, combining the modalities discussed here, has the potential to improve our understanding for appraising the pathology and interactions between heart failure and arrhythmia and can provide robust and validated outcomes in a variety of research settings. This review discusses the state of the art models, methodologies and techniques used in the evaluation of heart failure and arrhythmia and will highlight the benefits of using them in combination. Special consideration is paid to assessing the pivotal role calcium handling has in the development of heart failure and arrhythmia.

## Introduction

Heart failure and cardiac arrhythmias are intrinsically linked in a complex interplay of cause and effect. Cardiac arrhythmias can promote left ventricular systolic dysfunction through rapid ventricular rates which disrupt atrial and ventricular output (Prabhu et al., 2017). Moreover, heart failure is an independent risk factor for arrhythmogenesis, due to its deleterious impact on atrial remodelling (Heijman et al., 2014). Heart failure and arrhythmias have shared physiological and genetic causes. Furthermore, many of the methods and systems used to evaluate the electrophysiological changes that occur in cardiac arrhythmias are common to those used in heart failure research.

Advancements in medical therapies have led to the survival of patients with heart failure and arrhythmias for longer, increasing the prevalence of both conditions (Schmitt et al., 2009). Furthermore, in patients with inherited cardiac conditions, arrhythmias are common and represent a significant financial and clinical burden (Verheugt et al., 2010). The number of people living with chronic heart failure is increasing, estimated to be 64.3 million worldwide in 2020 (Groenewegen et al., 2020). An increased prevalence of atrial fibrillation (AF; 3.29% in 2016) in the United Kingdom over the past decade has compounded the issue, as it predisposes many to the development of heart failure and ischaemic stroke (Pozzoli et al., 1998; Eckardt et al., 2016; Adderley et al., 2019).

Research into the diagnosis, aetiology, prevention and treatment of cardiac arrhythmias has the potential to provide substantive clinical benefit to a significant proportion of the population and is particularly pertinent to those suffering from heart failure. Despite recent advances in cardiology, the mechanisms underpinning the multitude of different types of cardiac arrhythmias are still not fully understood.

Historically, researchers have been heavily reliant upon electrophysiological data obtained from clinical cases and animal models. Obtaining human experimental data, such as electrocardiograms and echocardiograms, is relatively inexpensive, available and non-invasive to the patient (Davie et al., 1996). However, the procurement and subsequent use of human tissue in cardiac arrhythmia research is often limited by stringent ethical approval and a lack of availability (Price, 2005).

Cardiovascular research requiring the use of animal models, such as mice, rabbit, goat and pig, is often highly invasive and consequently carries a substantial ethical burden. Moreover, although heart failure and cardiac arrhythmias have been successfully modelled *in vivo*, distinct interspecies differences in cardiac electrophysiology (e.g., heart rate of mice being approximately 10 times faster than in humans) limits the translation of these findings into the clinical setting. Recent developments in human-based methodologies, including induced pluripotent stem cells (iPSC) and computational cardiac modelling and simulation, present exciting prospects to supplement and augment experimental and clinical investigations (Rodriguez et al., 2015).

In the following text, we will outline many of the models and techniques most commonly used to evaluate cardiac arrhythmias in heart failure research. They are summarised in Table 1. For a broader description of the experimental models available for cardiac electrophysiology research, and their suitability for use in evaluating specific arrhythmogenic syndromes, the reader is directed to the excellently written review by Odening et al. (2021). Heart failure can arise from a multitude of aetiologies, including but not limited to inherited genetics, environment (including chemotherapy) and age (Ziaeian and Fonarow, 2016). While only present in a sub-group of patients with heart failure, this review will often use arrhythmias linked to genetic variation as a prime example, as this area of research has made significant advances within recent years.

**Table 1:** Methods used to evaluate cardiac arrhythmia in heart failure.

Approach	Method	Description	Invasiveness	Advantages	Limitations
*In vivo*	Electrocardiogram (ECG)	Measuring voltage versus time from electrodes placed on the skin	Non-invasive	Easy to perform Can be used to detect most sustained arrhythmias	Provides limited information on mechanism of arrhythmia Struggles to detect intermittent arrhythmias
*In vivo*	Echocardiography	Using sound waves to facilitate live imaging of the heart. This can be used to indirectly estimate measurements of the cardiac cycle	Non-invasive	Provides detailed structural information on the heart Relatively easy to perform	Cardiac cycle is estimated High interobserver variability
*Ex vivo*	Monophasic and transmembrane action potentials	The recording of action potentials from either a single or group of cardiomyocytes using intracellular and extracellular electrodes	Invasive/Non-invasive	Direct recoding of transmembrane voltage changes Can be recorded in freely beating heart/preparations Ideally suited for arrhythmia induction and testing	Low spatial resolution Direct electrode contact can damage tissue Hearts/tissue samples often require preparation, e.g., Langendorff perfusion
*Ex vivo*	Voltage and calcium optical mapping	Using voltage and/or calcium-sensitive dyes to analyse action potential propagation and calcium transients	Partially invasive	High spatial resolution allows visualisation of propagation patterns present in complex arrhythmias Enables the electrophysiological assessment of samples following electrical shocks which may be elicited to induce arrhythmogenesis or mimic defibrillation	Hearts/tissue samples often require preparation, e.g., Langendorff perfusion Motion artefacts can occur if samples are uncoupled High skill level required Dye toxicity and photobleaching
*Ex vivo/In vitro*	Patch clamping	Microelectrodes are used to interrogate membrane potential and ion current channel function in excitable cardiac cells and preparations	Invasive	Enables electrophysiological characterisation of a subset of individual ion channel(s) (voltage clamp) Enables the direct recording of action potentials (current clamp) Enables the comprehensive characterisation of electrophysiological events at a single-cell level under controlled conditions	High skill level required Cannot detect electrophysiological events related to re-entry Low throughput
*In vitro*	Multi-electrode arrays (MEA)	A surface containing embedded electrodes acts as a neural interface to assay the electrical activity of cultured cells	Non-invasive	High-throughput multiplexed reads Relatively unharmful to the cells, allowing experiments to be performed over a long period of time	Low spatial resolution An extracellular field potential is recorded rather than the action potential itself
*In vitro*	Intracellular calcium imaging	A fluorescent calcium indicator is either added to the cells or endogenously expressed to visualise calcium transients	Partially invasive	High spatial resolution allows assessment of intracellular calcium handling Can be performed in conjunction with voltage-sensitive dyes	Dyes can be toxic to the cells Skill required to determine the appropriate indicator/dye for imaging
*In silico*	Human-based computational models and simulations	Simulations using mathematical models of human cardiac pathophysiology yield high spatio-temporal resolution data, including time course of ionic currents, action potentials, calcium transients, conduction velocity and the ECG.	Non-invasive	Fast and cost-effective way of evaluating arrhythmias Can be used to generate predictions on arrhythmia mechanisms which would be imperceptible using solely experimental data	Can be reliant on experimental data Computational power is limited requiring researchers to balance the complexity of their model against its performance

## Models and Techniques used to Evaluate Arrhythmia in Heart Failure

### *In vivo*/*Ex vivo* Model Systems

#### Genetically Modified Animals

Following the pioneering work by Thomas and Capecchi (1987) on the site directed mutagenesis of mouse embryonic derived stem cells, genetically modified animal models have become a staple method commonly used in disease modelling. A myriad of genetic variations can be inserted into the embryos of animals to cause the overexpression, inactivation, conditional expression and modification of cardiac genes (Low et al., 2016). Modern genome editing techniques, such as clustered regularly interspaced short palindromic repeat (CRISPR) Cas9 editing, have allowed the engineering of animal genomes to be performed with unprecedented ease (Ran et al., 2013; Zarei et al., 2019). This has consequently led to the widespread use of genetically engineered animals in cardiovascular research (Ding et al., 2014; Carroll et al., 2016; Tessadori et al., 2018).

**Figure 1:**
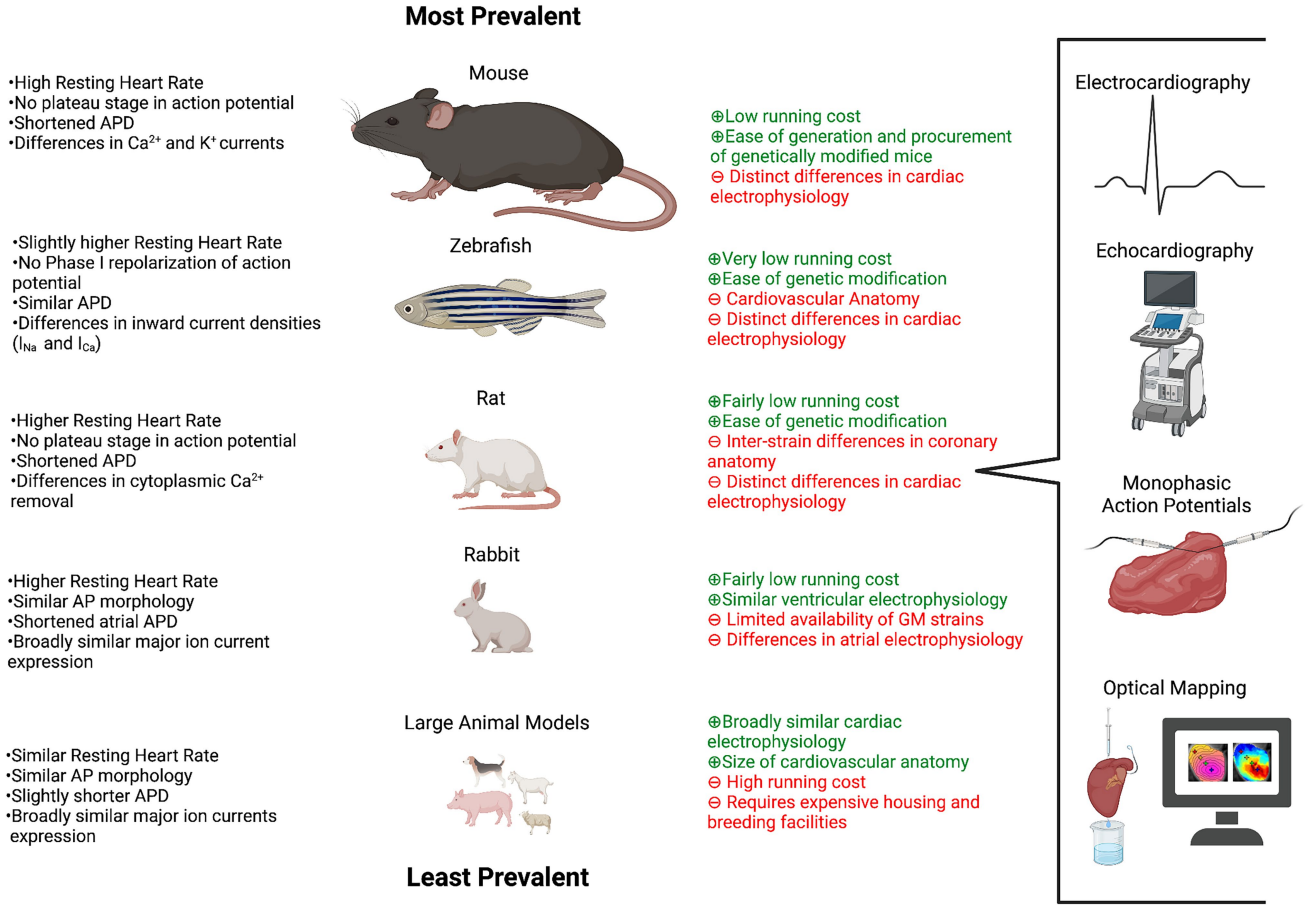
*In vivo* models used in cardiac arrhythmia and heart failure research. An outline of the animals used in arrhythmia and heart failure research, their electrophysiological similarities and differences in relation to humans, the advantages (green) and limitations (red) of their use and the techniques most commonly used in their evaluation. The size of the animal represents the prevalence of their use. Created with BioRender.com

A variety of genetically modified animals have been used to study heart failure and arrhythmias, including but not limited to rabbits, pigs, dogs and rats (Clauss et al., 2019). Figure 1 outlines the most commonly used animals in arrhythmia and heart failure research, their differences in electrophysiology in relation to humans and the methods used in their evaluation. The prevalence of large animals in arrhythmia research is comparatively small when contrasted to that of the mouse and zebrafish. Genetically modified mice, containing loss of function variants in the gap junction protein connexin43, frequently develop severe ventricular arrhythmias and have been used to model the arrhythmogenic substrates behind sudden cardiac death (Gutstein et al., 2001). Heart failure in *in vivo* models can be promoted in a variety of ways, including coronary artery ligation, aortic banding, chronic rapid pacing and isoproterenol infusion treatment (Chen et al., 2017a; Bosch et al., 2020). Many of these methods are detailed in Halapas et al. (2008) and can be performed on animal models possessing arrhythmogenic variants to study the complex pathogenesis of arrhythmias in chronic heart failure.

Channelopathies, such as long QT syndrome, have been recapitulated in mice, by the targeted mutagenesis of genes encoding subunits of inward rectifier potassium channels and *SCN5A* (Salama and London, 2007). However distinct differences in the ion channels predominantly responsible for cellular repolarisation in adult human and mouse cardiomyocytes exemplify how contrasts in interspecies cardiac electrophysiology limits the use of data obtained from such models (Wang et al., 1996).

Arrhythmogenic cardiomyopathies, often caused by genetic alterations, have been successfully modelled in genetically modified mice to assess the impact they have on the development of heart failure. The micropeptide phospholamban helps regulate intracellular calcium handling in cardiomyocytes by inhibiting the sarcoplasmic reticulum Ca^2+^-ATP-ase SERCA2 (MacLennan and Kranias, 2003). Pathogenic variants of the *PLN* gene have been linked to the development of arrhythmogenic cardiomyopathies and severe heart failure and have been successfully modelled in mice to assess arrhythmia susceptibility and response to standard heart failure therapy (Fish et al., 2016; Eijgenraam et al., 2020). Another example of cardiomyopathies being modelled in mouse models is evidenced in Geisterfer-Lowrance et al. (1996), where the group generated a *Myh6* p. Arg403Gln variant in the orthologous α cardiac myosin heavy chain (MHC) gene to explore the pathological effects of the variant in familial hypertrophic cardiomyopathy.

Common single nucleotide variants, identified in genome-wide association studies of AF and heart failure, are frequently found located in non-coding regions of the genome (Shah et al., 2020). The association between the variant and the disease is often unclear and can consequently require further elucidation using *in vivo* models. Genetic variants located in the 4q25 region, which lies adjacent to the *PITX2* gene, have been strongly linked to the development of AF (Gudbjartsson et al., 2007). The precise mechanism by which this genomic region affects the expression of *PITX2* and the development of AF remains cryptic. Genetically modified mouse models have proven powerful tools to validate disease association. The insertion of fragments of the 4q25 region attached to a reporter gene, into the genome of mouse embryos, has helped researchers explore the functional role variants in this cis-regulatory region have on cardiac development (Aguirre et al., 2015).

The use of genetically modified mouse models in arrhythmia and heart failure research poses a difficult challenge. Although mice and humans share approximately 85% sequence homology in protein coding regions, fundamental differences remain in the sequence composition of many key genes and their relative expression levels (Makałowski et al., 1996). Disparities in the compartment-specific expression of transient outward K^+^ current (I_to_), as well as voltage-gated sodium and calcium channel isoform expression causes stark differences in the formation of the cardiac action potential (Blechschmidt et al., 2008; Niwa and Nerbonne, 2010; Björling et al., 2013). Consequently, results obtained from mice often require translation when interpreted for humans (Tanner and Beeton, 2018).

The generation of humanised mouse models has attempted to mitigate differences in sequence homology through the replacement of the mouse gene with the orthologous human counterpart (Zhu et al., 2019). However, the complexity of gene expression regulation in higher eukaryotes makes precise transcriptional emulation difficult. The cost and time needed to generate genetically modified mouse models limits their use in investigating rare inherited variants associated with cardiomyopathies, arrhythmias and heart failure. Furthermore, genetically modified animal models struggle to emulate the environmental stressors and comorbidities of individuals with heart failure and arrhythmia and therefore struggle to capture the phenotypic spectrum of either disease (Colbert et al., 1997; Vakrou et al., 2018).

#### *Ex vivo* Cardiac Preparations

Pioneered by Oskar Langendorff, the retrogradely perfused heart allows prolonged experimental interrogation in a context independent of confounding non-cardiac organ function (Bell et al., 2011). The Langendorff heart is a cornerstone of basic cardiology research. It allows precise control of physiological and pharmacological interventions and facilitates programmed stimulation for arrhythmia induction. The effect that these interventions as well as genetic and environmental stressors have on the isolated heart, can be studied using several methodologies (section “Electrophysiological Study of *ex vivo* Model Systems”). The Langendorff heart is a non-working system which fails to fully recapitulate *in vivo* conditions due to its retrograde perfusion. The Langendorff model can be modified into the orthogradely perfused working model developed by Neely et al. (1967), to better characterise pump function. Further information on isolated heart models can be derived from Olejnickova et al. (2015).

Additional preparations of the animal heart have been developed from the whole heart to answer specific experimental questions. The innervated heart technique, originally developed by Ng et al. (2001) for use in the rabbit, has been applied in several animal models to enable study of autonomic influences on cardiac electrophysiology (Winter et al., 2018; Wang et al., 2019). Isolated atrial preparations enable detailed study of the atria and sinoatrial node without confounding ventricular influences, while slice and wedge preparations allow transmural properties of the mouse heart to be investigated (Lang et al., 2015; Holmes et al., 2016; Wen et al., 2018; Dong et al., 2019; Brennan et al., 2020).

### Electrocardiography in *in vivo* Model Systems

#### Fundamentals of Electrocardiography in Animal Models

The electrical changes that occur during the cardiac cycle can be plotted in a voltage versus time graph, commonly known as an ECG (Geselowitz, 1989). Recognisable complexes within the ECG, such as the P wave, QRS complex and T wave, correspond to the depolarisation of the atria (P) and ventricles (QRS) and the repolarisation of the latter (T). Willem Einthoven is credited with the invention of electrocardiography and the contemporary ECG (Barold, 2003). Historically, the use of electrocardiography was integral in defining many of the fundamental mechanisms behind clinically important arrhythmias (Fye, 1994). Today the technique underpins a significant proportion of modern cardiovascular research and is pervasively used to phenotype genetically modified animal models.

Heart rate and heart rate variability are two of the most important metrics determined from an ECG. Researchers use animal heart rates to characterise cardiac function in response to hemodynamic, pharmacologic and environmental stressors (Appel et al., 1989). Variation in heart rate, which arises from differential sinoatrial node stimulation, is influenced by the animal’s temperature, activity, stress level and sleep cycle (Thireau et al., 2008). It can be used as a measurement of how adaptive the animal is to cardiac stress, with a decreased variation in heart rate being linked to an increased risk of mortality following myocardial infarction (Kleiger et al., 1987). Intervals between recognisable complexes within the ECG, such as the QT, PR and RR, can be calculated and compared between animals with relative ease. Perturbation of such complexes can be used to identify structural abnormalities within the heart and can be prognostically important in the evaluation of heart failure and arrhythmia. For example, the RR interval can be plotted in Poincaré plots to identify the presence of AF (Park et al., 2009).

ECGs of genetically modified animals are often used to assess the pathogenic impact gene variants have on arrhythmogenesis. This has proven particularly pertinent when exploring variants associated with channelopathies and arrhythmogenic syndromes, such as those in the calcium ryanodine receptors (Zhao et al., 2015). Despite the overwhelming prevalence of the animal in cardiovascular research, the surface ECG of the zebrafish has and continues to be relatively underutilised. Further information on the practicalities of electrocardiography in zebrafish can be found in Zhao et al. (2019). The coming paragraphs will focus on electrocardiography in mice, due to their aforementioned common use in arrhythmia and heart failure research.

#### Experimental Methods for Electrocardiography in Mouse

The arrhythmias common in patients with heart failure are often sporadic and present inconsistently, therefore the induction of arrhythmias in mice is often required. Arrhythmias can be induced in a variety of ways including burst/S1-S2 pacing, intense endurance exercise and the administration of pro-arrhythmic agents (Schrickel et al., 2002; Spurney et al., 2011; Aschar-Sobbi et al., 2015). Electrocardiography can be performed on conscious or sedated mice, with the latter being disadvantageous as disruption of cardiac function can be caused by many of the commonly used sedatives (Chaves et al., 2003).

There are three established systems for the recording of ECGs from mice: non-invasive, tethered and implanted telemetry ECG (Ho et al., 2011). Non-invasive ECGs involve placing the mouse in a constraint so that three small surface electrodes make contact with the paws of the animal. As anaesthesia is not required and the technique is quick and easy to do, non-invasive electrocardiography facilitates “high-throughput” screening of mice; however, the technique is not suitable for long term ECG recordings.

Tethered electrocardiography involves attaching four small electrodes into the back of the mouse. The electrodes are tethered to a swivel device to enable unrestricted movement. ECGs can be recorded without the need of an often stress inducing restraining cage and for longer periods of time. General anaesthesia is however required to insert the electrodes into the mouse and may consequently lead to abnormal cardiac function. Mice must be monitored during the recording of the ECG to prevent agitation of the tethered electrode wires, limiting the use of the technique in long term experimental studies.

Implanted telemetry electrocardiography involves inserting a radio transmitter connected to two electrodes into the mouse. Signals are received wirelessly by a nearby amplifier and computer system. The technique enables ECGs to be recorded over a prolonged continuum, enabling heart rate variability to be monitored and arrhythmia frequency to be calculated (Knollmann et al., 2003). Implanted telemetry electrocardiography allows researchers to determine whether arrhythmic events were responsible for cause of death. The surgery required for implanted telemetry ECGs poses significant risk of mortality and morbidity to the mouse (Schuler et al., 2009). A recovery period is required following the surgery, making the technique more suited to use in long term electrophysiological studies.

#### Utility of Electrocardiography in Mouse

Electrocardiography is often described as the “gold standard” technique for the electrophysiological analysis of the heart. It lacks the spatio-temporal resolution afforded to optical mapping but exceeds in its capacity for comprehensive *in vivo* characterisation. Alternative methods, such as echocardiography, which indirectly determines heart rate, provide limited information on the electrophysiology of the cardiac cycle and is unable to discriminate between sinus and ectopic heartbeats. This consequently constrains its use in the evaluation of complex ventricular arrhythmias associated with chronic heart failure. Echocardiography is extensively used in cardiovascular research to characterise the structural cardiac phenotype of genetically modified animal models; however, due to its restricted use in arrhythmia research, it will not be covered in detail in this review. Further information on the role echocardiography has in basic and clinical cardiovascular research can be obtained from Scherrer-Crosbie and Thibault (2008).

Comparing ECGs generated from mice to those derived from humans is not straightforward but is essential when assessing arrhythmogenesis of heart failure models. Bazett’s formula, which is commonly used to equate QT intervals measured from contrasting heart rates, fails to account for the differences present in mice sedated by certain anaesthetics (Boukens et al., 2014). The distinct differences in the cardiac electrophysiology of mice and humans are evidenced by both the heart rate and action potential duration (Kaese and Verheule, 2012). Further contrasts are evidenced by morphological changes in complexes of the ECG, such as an ambiguous ST segment and an additional J wave. The J wave arises in the mouse (and other rodents) ECG due to the lack of a plateau phase in the action potential, meaning early repolarisation is visible as a positive deflection shortly after the QRS complex (Offerhaus et al., 2021). It is for this reason also that the mouse ECG has a less pronounced T wave.

As well as morphological changes present in the sinus rhythm of mice and humans, patho-anatomical changes can cause varying responses in the ECG of humans and mice. Acute myocardial ischemia is represented by the elevation of the ST segment in humans, while in mice it is conversely shown as a reduction in S wave amplitude followed by an abnormal J wave and inverted T wave (Janse, 1986; Gehrmann et al., 2001). The potential of the surface ECG in mice is largely restricted by the size of the animal. Although not limited to its use in mice, electrocardiography is still performed comparatively little in larger, more electrophysiologically analogous mammals, such as pigs and dogs. This is mainly due to the cost associated with the animals housing and upkeep and the more stringent ethical restrictions covering their use in research.

Further to surface ECG recording, methods have been developed to directly record electrical activity of the *in vivo* mouse heart at the epicardial surface (*via* an open torso approach) and intracardially (*via* transvenous catheters; Berul et al., 1996; VanderBrink et al., 1999). Such approaches are advantageous over the surface ECG as they enable recording of an ECG to be taken under programmed stimulation elicited to unearth arrhythmia in animal models with altered myocardial structure (Maguire et al., 2000; Saba et al., 2000; Sawaya et al., 2007). However, they are limited by the relatively low spatio-temporal resolution associated with indirect extracellular ECG recordings.

### Electrophysiological Study of *ex vivo* Model Systems

#### Monophasic and Transmembrane Action Potential Recordings

Electrode-based methods allow the recording of action potentials from the isolated heart and other *ex vivo* cardiac preparations. Intracellular microelectrodes can be used to record transmembrane action potentials from a single cell within the intact preparation or indeed from isolated cardiomyocytes (section “Cellular Systems: Primary Cells”). By using one electrode in the intracellular space and another extracellular electrode, the difference between the two signals facilitates the recording of the transmembrane action potential (Holmes et al., 2016).

Larger electrodes (>.1 mm diameter), positioned firmly against cardiac tissue, can be used to record extracellular activity originating from several cells (Kirchhof et al., 1998; Fabritz et al., 2003; Iravanian et al., 2020). These recordings are known as monophasic action potentials (MAPs) and are routinely recorded from Langendorff perfused animal hearts to directly assess cardiac electrophysiology. Freundt et al. (2019) recorded MAPs from rabbits following treatment with the histone deacetylase inhibitor, entinostat, to demonstrate that the drug could prevent heart failure associated early after depolarisations (EADs) and structural remodelling. The setup required to record these signals consist of a proximal and distal electrode, neither of which crosses the cellular membrane. The exact mechanisms behind the origin of monophasic action potential recordings are not fully understood; however, they are thought to rely on proximal inactivation of one part of the tissue (Franz, 1991; Tse et al., 2016).

#### *Ex vivo* Optical Mapping

##### Overview

Electrode techniques inherently have low spatial resolution due to the physical constraint of electrode placement. Cardiac excitation however involves the coordinated (or uncoordinated in the case of some arrythmias) propagation of action potentials across the tissue. Furthermore, tissue heterogeneities, such as activation or repolarisation dispersion and areas of ectopic activity, are often fundamental mechanisms for arrythmia induction in patients with heart failure. Therefore, higher spatial resolution mapping techniques are required for mechanistic research of cardiac preparations. These include multielectrode array techniques (section “Multi Electrode Arrays”) and optical mapping.

Cardiac optical mapping is a method used to investigate the electrical properties of cardiac tissue preparations through the excitation of fluorescent dyes (Zhang et al., 2016; O’Shea et al., 2020). Staining with voltage-sensitive indicators, such as potentiometric Di-4-ANEPPs, enables adjustments in membrane potential to be monitored with greater spatial resolution than electrode-based methods. Calcium-sensitive indicators are utilised to visualise intracellular calcium handling. Furthermore, co-staining with voltage and calcium-sensitive indicators allows concurrent mapping of both calcium transients and action potential propagation (O’Shea et al., 2019b). The information in the following section pertains to the optical imaging of *ex vivo* heart samples, although much of it remains highly relevant to the optical imaging of *in vitro* models, discussed in section “Calcium Imaging in *in vitro* Model Systems.”

Optical mapping was first developed to study the membrane potentials of neuronal cells by Salzberg et al. (1973). The extension of its use to cardiac research by Salama and Morad (1976), enabled the electrophysiological characterisation of cell samples which were previously awkward to assay by traditional microelectrode-based methods. The further development of optical mapping techniques enabled the imaging of retrogradely perfused animal hearts and other *ex vivo* preparations (Salama and Choi, 2000).

Optical mapping has become a routinely performed experimental technique used to evaluate arrhythmogenesis in isolated perfused hearts and *ex vivo* cardiac preparations. The basic setup for the optical mapping of an *ex vivo* cardiac tissue preparation consists of three main parts: a sample to image, equipment designed to elicit fluorescent excitation and a detector for the recording of spectral emission. Optical mapping of cardiac tissue samples facilitates the visualisation and recording of action potential propagation and duration. The significantly greater spatial resolution afforded to optical mapping has enabled the visualisation of complex propagation patterns present during cardiac arrhythmia and has helped to identify both the macro- and micromechanisms behind them (Girouard et al., 1996). Optical mapping has proven particularly pertinent in the research of re-entrant arrhythmias enriched in patients with chronic heart failure, such as atrial and ventricular fibrillation, where it has enabled the visualisation of spiral waves in isolated epicardial muscle (Pertsov et al., 1993; Masarone et al., 2017).

Optical mapping has been used to investigate mechanisms behind atrial fibrillation in age-related heart failure with preserved ejection fraction (Mesquita et al., 2020). The group used *ex vivo* preparations derived from aged rats prone to heart failure with preserved ejection fraction to demonstrate slowed conduction velocities and perturbed β-adrenergic response. In contrast to microelectrode-based monitoring, the output of cardiac optical mapping remains broadly unaffected by high-voltage shocks. This allows the electrophysiological response of samples to be determined following the elicitation of electrical shocks designed to mimic defibrillation or induce arrhythmogenesis (Chattipakorn et al., 2001; Fast and Cheek, 2002).

##### Limitations of Optical Mapping

Optical mapping however has its limitations. Contractile movements from the cardiac sample can distort pixel imaging and create artefacts in the measured signal. Motion suppression can be achieved using uncoupling agents, such as blebbistatin. However, although useful, uncoupling agents can cause significant disruption to the electrophysiology of the cells and can shroud important interactions that occur due to mechano-electrical feedback. Significant prolongation of the action potential and an increase in ventricular fibrillation have been reported following the treatment of rabbit hearts with blebbistatin, demonstrating possible limitations with its use (Brack et al., 2013; Kappadan et al., 2020). Other reports however have suggested that blebbistatin exerts little direct influence on cardiac electrophysiology (Fedorov et al., 2007).

Methods have therefore been developed to image mechanically coupled cardiac preparations. Ratiometric optical mapping involves recording signals using two different excitation or emission wavelengths. In this approach, two signals are recorded which are differentially altered by calcium concentration or voltage, but similarly corrupted by motion. Therefore, the ratio between the signals can be used to mitigate the impact of motion artefacts (Knisley et al., 2000; Bachtel et al., 2011). Sophisticated motion tracking algorithms, developed to reduce noise in mechanically coupled hearts, can be used effectively in conjunction with ratiometric optical mapping to further reduce motion artefacts (Rodriguez and Nygren, 2014; Garrott et al., 2017; Christoph and Luther, 2018). Analysis of optical mapping data requires highly specialised algorithms. This originally restricted use to laboratories that could develop these in-house. Recently however the emergence of open-source, versatile and high-throughput software by several different laboratories has meant that this is no longer a significant limitation (Gloschat et al., 2018; O’Shea et al., 2019a; Tomek et al., 2021).

### *In vitro* Model Systems

#### Cellular Systems: Primary Cells

*In vitro* models consisting of excitable, functional primary cardiomyocytes can be derived from enzymatically treated cardiac tissue using Langendorff perfusion, the newly developed Langendorff-free method and the so-called “chunk method”, which is commonly used on isolated human heart tissue (Yue et al., 1996; Workman et al., 2001; Louch et al., 2011; Holmes et al., 2021). Cell culture models consisting of primary cardiomyocytes offer an easily manipulated and physiologically relevant model for heart failure and arrhythmia research. The cells used are often derived from the explanted hearts of patients with end-stage heart disease (Zhang et al., 2021). Such models have proven particularly useful in investigating the fundamental cellular mechanisms behind arrhythmia due to physiological ion channel expression within the cells. Pérez-Hernández et al. (2016) were able to demonstrate that increased expression of *PITX2c*, which is commonly seen in the atrial appendages derived from patients with AF, could alter the densities of the slow delayed rectifier potassium channel (I_Ks_) and L-type calcium channel (I_CaL_) in human atrial myocytes (Gudbjartsson et al., 2007).

The inaccessibility of healthy human reference tissue and the limited proliferation potential of the cells derived in culture have however impeded the widespread use of primary human cardiac cells in heart failure research (Ikenishi et al., 2012). Primary cardiac preparations derived from small laboratory animals, such as mice and rats, are comparatively abundant and consequently their use in arrhythmia and heart failure research is common. Non-human primary cardiomyocytes were first used to study the effects that inotropic agents had on the membrane potential of single cells (Iijima et al., 1985). Patch clamping, a technique used to record the membrane voltage and ion channel activity in isolated cells or tissue sections, was often utilised in such experiments (section “Patch Clamp”). Advancements in the optical imaging of calcium- and voltage-sensitive dyes (section “Calcium Imaging in *in vitro* Model Systems”) expanded the utility of primary non-human cardiomyocyte models in arrhythmia research and enabled, for the first time, the visualisation of spontaneous re-entrant waves in myocyte monolayers (Bub et al., 1998).

**Figure 2:**
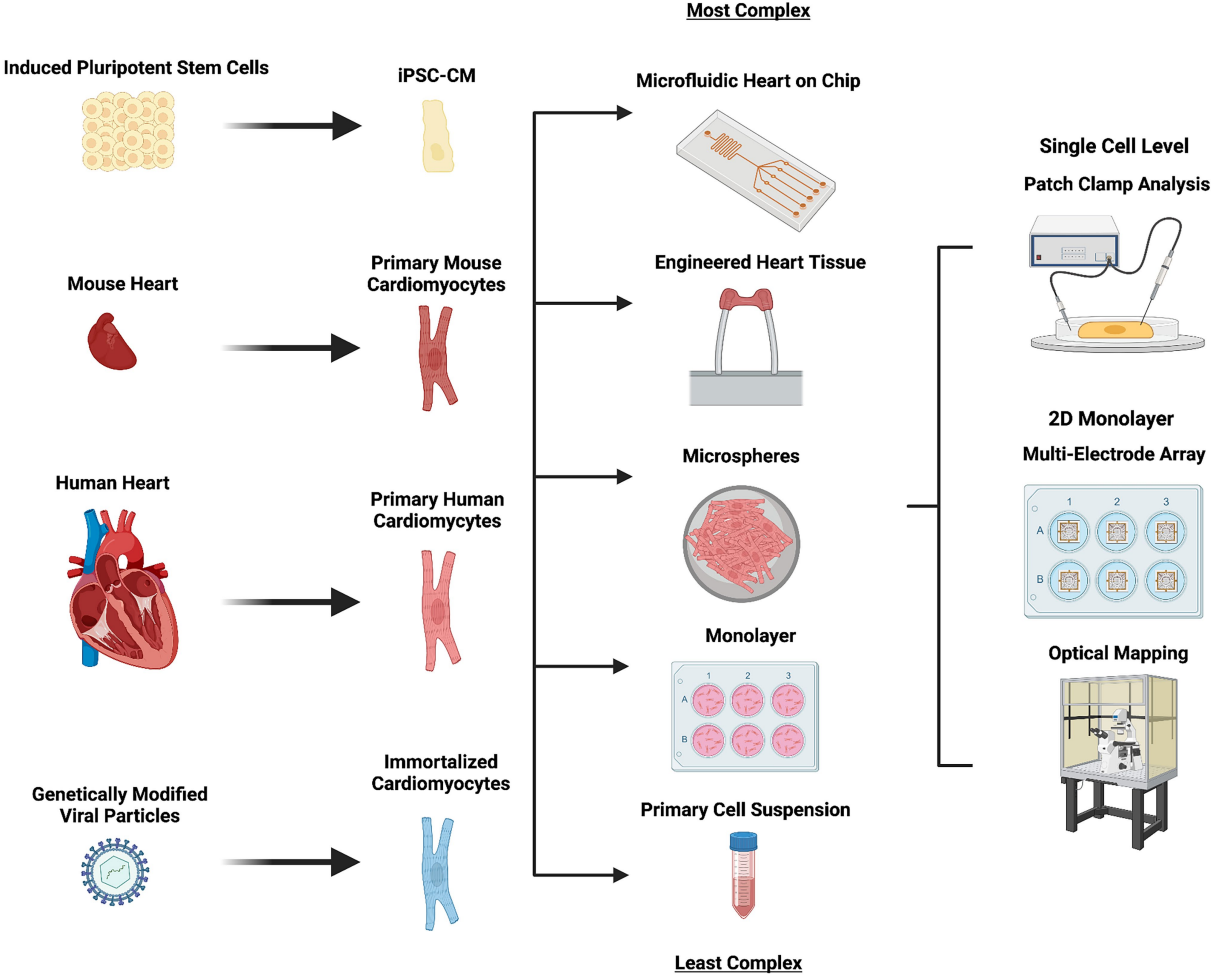
*In vivo* models used in cardiac arrhythmia and heart failure research. An outline of the *in vitro* cell models used in arrhythmia and heart failure research, how they are derived (left), the format in which they can be used (middle right) and the techniques most commonly used in their evaluation (right). Complexity of the model used increases from bottom (primary cell suspension) to top (microfluidic heart on chip). Created with BioRender.com

The development of 3D engineered heart tissue models from primary neonatal rat cardiomyocytes has allowed greater phenotypic maturation and the generation of a system particularly well suited to cardiotoxicity drug screening (Krause et al., 2018). Significant electrophysiological differences in action potential duration and intracellular calcium handling in human and rodent species however continues to limit the validity of results obtained using animal cardiomyocytes (Figure 2).

Cardiovascular research using human and non-human primary cardiomyocytes is hampered by the cells lack of propensity for proliferation. In spite of this, they have been used with great effect in understanding the electrophysiological changes that occur during heart failure. Maltsev et al. (2007) demonstrated that the cardiomyocytes derived from failing human and dog hearts were prone to early after depolarisations due to increased variation in action potential duration. An increase in late sodium current (I_Na_) activity was identified as a potential cause, with inhibition of the current reducing action potential duration variability and the presence of early after depolarisations.

#### Cellular Systems: Immortalised Cardiac Cells

Immortalised cardiomyocyte cell lines can be generated from human and non-human cardiac tissue. They can be readily expanded *in vitro*, theoretically circumventing one of the major limitations associated with primary cardiac cells (Davidson et al., 2005). In reality, the proliferative capacity of immortalised cardiomyocytes can limit their use as a viable cardiac model. This is due to the instability of their myofibrils, which are continually undergoing disassembly during cell division (Ahuja et al., 2004; Onódi et al., 2022). Immortalised cardiac cell lines can be generated through the ectopic expression of the oncogene *SV40*, which allows mitotically arrested cells to re-enter the cell cycle and proliferate (Ramkisoensing et al., 2021).

HL-1, a renowned mouse cardiac (atrial) cell line, has been successfully used to model the effects of structural and electrical remodelling in AF development (Wiersma et al., 2017; Zhang et al., 2018). More recently, it has been used to investigate the effect overexpression of microRNAs (mRNAs) have in patients with heart failure with reduced ejection fraction and AF (Garcia-Elias et al., 2021). The group demonstrated that exposure of HL-1 cells to the mRNAs identified in patients with heart failure and AF caused disruption to calcium handling and cell to cell communication.

One major limitation of immortalised cardiac cells is that the uncontrolled expression of oncogenes can cause the generation of a population of cells with desynchronised cell cycles. Over time, this can lead to a heterogenous population of cells with disparate electrophysiological and functional properties. The development of conditionally immortalised cell lines, in which the SV40 oncogene is under the control of an inducible promoter, has partially addressed this limitation and has enabled the generation of models with greater electrophysiological maturity and homogeneity (Liu et al., 2018). The non-cardiac cell line human embryonic kidney 293 is used in cardiac research to explore the effect pathogenic variants have on the activity of specific ion channels. Pathogenic variants of ion channel genes can be transiently expressed in the cells to elucidate cellular mechanisms behind cardiac disease (Prakash et al., 2021). The reader is directed to Odening et al. (2021) for a more detailed description on the role such cells play in cardiac electrophysiology research.

#### Cellular Systems: Induced Pluripotent Stem Cells

##### Generation of Cardiomyocytes From Induced Pluripotent Stem Cells

Following the pioneering work by Takahashi and Yamanaka (2006) in identifying transcription factors capable of inducing pluripotency in somatic cells, cellular reprogramming technology has revolutionised disease modelling. The generation of induced pluripotent stem cell (iPSCs) lines from genetically diverse individuals has enabled researchers to explore the impact common and rare genetic variants have on complex disease.

The relative ease in which genetic engineering can be performed on iPSCs is unparalleled in primary and immortalised cell lines. This can consequently facilitate the “high-throughput” screening of pathogenic variants. Cultures of iPSCs can be differentiated into cardiomyocytes by the manipulation of the Wnt signalling pathway. This allows the generation of a variety of cardiac cell types, including ventricular, atrial and nodal cardiomyocytes (Burridge et al., 2014; Schweizer et al., 2017; Cyganek et al., 2018).

In contrast to primary and immortalised cardiac cells, iPSCs act as both a renewable and reliable source of cells. Free from the ethical restrictions concomitant with embryonic stem cells and capable of being derived from individuals that vary in age, sex, race and disease state, the versatility afforded to iPSCs has led to their routine use in arrhythmia and heart failure research.

##### Cardiovascular Research Using iPSC-Derived Cardiomyocytes

The adoption of induced pluripotent stem cell models into arrhythmia and heart failure disease modelling has not come without challenges. Inefficient differentiation protocols yielding heterogeneous and often phenotypically immature cardiac cells has hindered the use of iPSCs in the modelling of many complex cardiovascular diseases (Goedel et al., 2017). Despite this, iPSC-derived cardiomyocytes (iPSC-CMs) have been successfully used to model channelopathies including long QT and Brugada syndrome (Savla et al., 2014). The monogenic aetiology of many channelopathies means that phenotypic variation can often be adequately assessed in single-cell assays. This circumvents the need for vast quantities of homogenous cardiac myocytes.

In contrast to some of the other physiological properties of the iPSC-CM, the activity of many of the key ion currents (inward sodium current, inward calcium current, delayed rectifier current, transient outward current) is broadly similar to human adult cardiomyocytes (Knollmann, 2013). Itzhaki et al. (2011) used multi-electrode arrays and patch clamping (sections “Patch Clamp” and “Multi Electrode Arrays”) to analyse iPSC-CMs derived from a patient with congenital long QT syndrome. The patient possessed a missense variant in the potassium voltage-gated ion channel subunit gene *KCNH2*. The cells demonstrated EADs and prolonged action potentials, due to a reduction in rapid delayed rectifier (I_Kr_) current activity.

Genetic variants identified in patients with cardiomyopathies and/or arrhythmias have been successfully modelled in iPSC-CM to investigate the molecular mechanisms behind their pathogenesis. Mutations within the *TTN* gene, that encodes the sarcomeric protein titin, are strongly linked to the development of familial dilated cardiomyopathy and atrial fibrillation (Herman et al., 2012; Choi et al., 2018). They have been successfully modelled in iPSC-CM to deepen our understanding of the pathogenic impact titin variants have on sarcomere organisation and calcium handling (Schick et al., 2018).

##### Challenges of iPSC-Derived Cardiomyocytes

The greatest challenge associated with the widespread employment of iPSC models in cardiovascular research remains the phenotypic immaturity of the derived cardiac cells. This is evidenced by the automaticity, reduction of inwardly rectifying potassium current (I_K1_) density and relatively positive diastolic membrane potential present in many populations of iPSC-CM (Goversen et al., 2018). The problem is further exacerbated when considering the age-related dependency of many cardiovascular diseases and arrhythmogenic syndromes. There is a myriad of methods used to enhance maturation of iPSC-CM. These can range from mechanical and electrical stimulation of the cells to the construction of 3D organoids. Many of these methods are comprehensively described in Machiraju and Greenway (2019).

Current differentiation protocols can generate cells that demonstrate tissue-specific expression of atrial, ventricular and nodal ion channels, transporters and connexins (Schweizer et al., 2017; Cyganek et al., 2018). Current protocols, however, often generate mixed populations of cells and are to our knowledge unable to specify the generation of cells from either the left or right chambers of the heart. This is of particular importance when considering the compartmental origin of the different types of heart failure. The optimisation of cellular differentiation protocols is often limited by the onerous and expensive nature of cellular differentiation and characterisation. The recent incorporation of genetically encoded calcium sensors (section “Genetically Encoded Calcium Indicators”) into commonly used iPSC lines has helped ameliorate this by facilitating high-throughput phenotypic screening of iPSC-CM following cellular differentiation (Chen et al., 2017b).

Re-entrant arrhythmias commonly seen in patients with heart failure often present due to structural differences in the 3D anatomy of the heart. This is challenging to model *in vitro* in 2D monolayers. The integration of iPSC-derived cardiac cells in co-culture and three-dimensional culture systems can provide models that demonstrate significantly greater phenotypic maturity and physiological relevance (Lemoine et al., 2017). However, they are still some way off recapitulating the intricacies of the cardiac micro-anatomy and intra-chamber regional variability which are important to both arrhythmia and heart failure development (Holmes et al., 2016). Furthermore, pathophysiological stressors including diabetes, hypertension, hypoxia, ageing, obesity and reduced cardiac blood flow, which act as major drivers for arrhythmogenesis and heart failure, are difficult to recapitulate, even in 3D iPSC-CM cultures (Yildirir et al., 2002; Lau et al., 2013; Chow et al., 2014; Pathak et al., 2015; Morand et al., 2018).

##### Emerging Strategies to Improve iPSC-Derived Cardiomyocyte Models

In recent years, an amalgamation between iPSC disease modelling and tissue engineering has fathered the generation of three-dimensional iPSC-CM models, such as cardiac microspheres and engineered heart tissue (Figure 2; Schaaf et al., 2011; Beauchamp et al., 2015). Such models are capable of demonstrating improved intracellular calcium handling and I_K1_ current densities (Buikema et al., 2013; Amano et al., 2016; Silbernagel et al., 2020). A comprehensive description of three-dimensional *in vitro* cardiac models is beyond the scope of this review, the reader is directed to Salem et al. (2021) for a current report describing such models.

The incorporation of co-culture and three-dimensional culture systems into microfluidic “heart on chip” platforms is an exciting prospect. In-built optical and electrical sensors allow data to be generated on calcium handling and contractility (Cho et al., 2020). Furthermore, microfluidic chips enable greater control over culture conditions, such as pH and substrate stiffness, with future iterations possibly permitting researchers to adjust parameters to consider pathophysiological stressors important in heart failure, including hypoxia and reduced blood flow (Beauchamp et al., 2020).

As is the case with primary and immortalised cell lines, the maintenance cost required for the use of iPSCs in arrhythmia and heart failure research is substantially lower than that of maintaining *in vivo* models, such as mice and zebrafish. Pathological variants of genes that cause embryonic lethality in mouse models can be modelled in iPSC models without the design and generation of complex conditional expression systems (Nishii et al., 2014). Despite this, there is scepticism about the *in vivo* reproducibility of experimental data derived from iPSC models. Presently, validation of such experimental data is often required in small rodent animals. The development of more efficient differentiation protocols and maturation strategies will likely facilitate the generation of iPSC-derived cardiomyocytes that are phenotypically much closer to adult cardiac myocytes. Furthermore, future iterations of co-culture model systems will provide greater accuracy in replicating the cardiac micro-anatomy.

### Electrophysiological Study of *in vitro* Model Systems

#### Patch Clamp

##### Overview

Patch clamping is the definitive technique used to study ionic currents and membrane potential in tissue samples, isolated cells and expression systems. Patch clamping has and continues to be the gold standard for studying ion channel activity in excitable cells including cardiomyocytes and neurones (Guinamard et al., 2004; Alloui et al., 2006). There are a myriad of patch clamping setups used to monitor the electrophysiology of cells under a variety of controlled conditions. The reader is directed to Kornreich (2007) for an in-depth description of patch clamping setups and their suitability in addressing specific research questions.

Patch clamping can be broadly separated into two types. Voltage clamping involves “clamping” cardiac myocytes at different defined membrane potentials, in order to elicit specific currents of interest which can then be recorded. This often takes place in the presence of numerous pharmacological agents which block other ion channels allowing for the isolation of a single current. Conversely, in the current clamp setup, the researcher controls the current being injected into the cell and records the membrane potential. This is usually in the form of an action potential. Both setups are routinely used in heart failure and arrhythmia research to understand the impact genetic variants, drug treatment and hypoxia have on ionic current, action potential morphology and resting membrane potential (Chavali et al., 2019; Plant et al., 2020).

##### Patch Clamping in Arrhythmia and Heart Failure Research

Patch clamping is used in heart failure research to investigate cardiac electrical remodelling in a variety of *in vitro* model systems including primary, immortalised and iPSC-derived cardiomyocytes. Hallmarks of arrhythmia in heart failure, which can be detected in *in vitro* cardiac cell models using patch clamping, include but are not limited to depolarised resting membrane potentials (largely due to a reduction in I_K1_), delayed after depolarisations (due to spontaneous Ca^2+^ leak from the SR and activation of the depolarising sodium-calcium exchanger), early after depolarisations (subsequent to reactivation of I_CaL_ and possibly I_Na_), prolongation of the action potential duration [primarily dependent on a decrease in major repolarising currents including I_to_, I_Ks_ and I_Kr_, but also due to enhanced late sodium current (I_NaL_)], ectopic automaticity, sinus node dysfunction and calcium handling disruption, recently reviewed in full by Husti et al. (2021). That said, ion channel remodelling in heart failure can display significant variation between individuals likely dependent on the different underlying origins and types of heart failure and the extent of disease progression. Shemer et al. (2021) used patch clamping techniques to interrogate the electrophysiology of iPSC-CM derived from two patients with *LMNA*-related dilated cardiomyopathy. Patients with *LMNA*-related dilated cardiomyopathy are at risk of severe heart failure and sudden cardiac death (Pasotti et al., 2008). The group identified delayed and early after depolarisations, as well as prolonged action potential durations in the iPSC-CM. This consequently increased our understanding of the mechanisms causing severe ventricular arrhythmias in patients with *LMNA*-related dilated cardiomyopathy.

Patch clamping is a technique that offers researchers unparalleled interrogation of the intracellular electrophysiology of cardiac cell models. However, patch clamping is relatively low throughput, with recordings being obtained from a single cell for a short period of time. The technique is highly skilled and consequently requires extensive time to master. Finally, there is still considerable subjectivity involved in choosing which cell to record from. This is exacerbated when patching iPSC-CM which are often heterogeneous, varying in shape, size and electrophysiological phenotype. Many of these limitations are being overcome using easy-to-handle automated patch clamp systems, which can improve throughput and standardisation and are comprehensively described in Suk et al. (2019), Obergrussberger et al. (2021), and Bell and Fermini (2021).

#### Multi-Electrode Arrays

##### Overview

Multi-electrode arrays (MEAs) are a non-invasive methodology used to assess the regional electrophysiology activity/heterogeneity in multicellular preparations. They have been used to measure electrical propagation in primary cardiac tissue, cultured monolayers of neonatal cardiac myocytes, immortalised cardiac cell lines and iPSC-derived cardiomyocytes (Wells et al., 2019). Cells are cultured on a surface embedded with dot-like electrodes to monitor regional extracellular field potentials at different points across the preparation, over a prolonged period (Spira and Hai, 2013). Changes in extracellular voltage occur due to the propagation of a spontaneous or stimulated action potential through the cell monolayer. The recorded field potential can be subsequently used to directly measure or estimate key electrical parameters including activation patterns, conduction velocity, spontaneous beating frequency, field/action potential duration and field/action potential amplitude (Halbach et al., 2003; Wells et al., 2019). Further information on the fundamentals behind MEA technology and the practicalities behind its use with cardiac cell types is beyond the scope of this review but can be obtained from Clements (2016) and Kussauer et al. (2019).

##### MEAs in Arrhythmia and Heart Failure Research

The adoption of MEAs into cardiac electrophysiology research has occurred relatively recently, with systems previously being designed for use in assessing the electrical activity of neural networks (Erickson et al., 2008). MEAs are broadly used on *in vitro* cell models to provide an overall assessment on the electrophysiological state of cardiomyocytes, in a way not dissimilar to the use of ECGs in *in vivo* models. MEAs have been used to ascertain the effectiveness of anti-arrhythmic therapies. For example, a study by Kim et al. (2022) used MEAs to evaluate the potential use of cardiac radioablation in the treatment of refractory ventricular arrhythmias, commonly seen in patients with heart failure (Peichl et al., 2021). The group monitored the electrical activity of iPSC-CM following irradiation, to further understand the electrophysiological response of the cells to the treatment. Despite this, MEAs are currently most often employed in assessing cardiotoxicity of pharmacological therapeutics. The effect the drug has on the field potential can be translated onto the action potential and subsequently used to predict *in vivo* cardiotoxicity (Braam et al., 2010; Colatsky et al., 2016; Tertoolen et al., 2018). Further information on the role MEAs play in *in vitro* drug research is beyond the scope of this review but can be obtained from Andrysiak et al. (2021). The main advantages of MEAs are that they are high-throughput and allow experimentation over prolonged periods, unlike patch clamping based methodologies. However, they are unsuitable for assessing the electrophysiology of single cells and lack the signal complexity afforded to intracellular interrogation. An exciting prospect for the future is the amalgamation of MEA technology into microfluidic heart on chip models. This may allow the electrophysiological response of cardiac cells to be monitored under pathological conditions associated with heart failure, such as hypoxia and hypokalaemia (Liu et al., 2020).

#### Calcium Imaging in *in vitro* Model Systems

Calcium (Ca^2+^) flux is the principal determinant of contraction in cardiac myocytes (Bers, 2002). Intracellular calcium handling underlies excitation–contraction coupling and is commonly perturbed in patients with cardiac arrhythmia and end-stage heart failure (Gwathmey et al., 1987; Ter Keurs and Boyden, 2007). Detailed information regarding the role intracellular calcium handling plays in cardiac arrhythmia and heart failure is beyond the scope of this review but is excellently summarised by Landstrom et al. (2017). Disruption to calcium handling can be caused by a number of mechanisms. Genetic variants of key ion channels, such as Ryanodine receptor 2, are one such example and can predispose individuals to arrhythmogenic syndromes and heart failure (Swan et al., 1999; Dridi et al., 2020).

The most dynamic and recognisable process in intracellular calcium handling is the release and subsequent re-sequestration of Ca^2+^ by the sarcoplasmic reticulum. This is known as a whole-cell calcium transient and commonly occurs prior to the contraction of a cardiac myocyte. It can be measured in primary, immortalised and iPSC-derived cardiac cell models. The spatial analysis of calcium transient kinetics has been used to explore mechanisms behind pathogenic variant driven arrhythmias and chronic heart failure in *in vitro* cell models. Lehnart et al. (2006) demonstrated diastolic Ca^2+^ leak from the sarcoplasmic reticulum of cardiomyocytes derived from mice deficient in calstabin-2, a protein key to ryanodine receptor 2 stabilisation, while Yin et al. (2014) used calcium imaging to elucidate the effect arrhythmogenic calmodulin variants had on intracellular calcium handling. It is worth noting that calcium imaging is a skilled technique, where careful consideration of the appropriate indicator is required.

##### Calcium Dyes and Indicators

###### Chemical Calcium Indicators

A range of light emitting dyes have been used to image Ca^2+^ in *in vitro* cardiac models. The dyes can be broadly categorised as being ratiometric or non-ratiometric. Ratiometric dyes display a shift in excitation or emission spectra following the binding of Ca^2+^. The ratio between the spectra allows the calculation of the absolute concentration of Ca^2+^ which is pertinent when measuring the amplitude of Ca^2+^ transients (Van Meer et al., 2016). An increase in fluorescence from non-ratiometric dyes corresponds to an increase in the relative concentration of cytosolic Ca^2+^. As no spectral shift is observed when a non-ratiometric dye is bound to Ca^2+^, variability in dye loading and cell permeability can cause a greater susceptibility to inter-assay variation. While ratiometric dyes are advantageous in capturing contractile behaviour for arrhythmia research, many imaging setups do not support their use (Jaimes et al., 2016).

Tetracarboxylate-based probes, synthesised by Tsien (1983), acted as blueprints for the fabrication of contemporarily used ratiometric and non-ratiometric calcium probes. Cyclically fluorescent and capable of traversing the sarcolemma, the dyes enabled the prolonged imaging of intracellular Ca^2+^ in cells derived from myocardial tissue without the inconvenience of cellular microinjection. Further iterations of the dyes led to the development of the 1,2-bis(2-aminophenoxy)ethane-*N,N,N′,N′*-tetraacetic acid (BAPTA) based probes fura-1 and fura-2. The BAPTA based dyes resolved limitations associated with previous tetracarboxylate-based probes, including narrow excitation/emission spectra and autofluorescence. Furthermore, they provided additional benefits including improved Ca^2+^ selectivity and the use of ratiometry (Grynkiewicz et al., 1985).

The synthesis of fluorescent indicators based on the chromophores rhodamine and fluorescein by Minta et al. (1989) facilitated the imaging of cytosolic Ca^2+^ transients at greater resolutions. Probes derived from these chromophores, such as rhod 1 and fluo 1, are non-ratiometric and display a lower affinity for Ca^2+^. This consequently confers improved dynamic range and increased sensitivity during calcium imaging. Properties, such as these, make the dyes particularly suitable for the imaging of ephemeral Ca^2+^ flux and intracellular diastolic calcium removal (Lock et al., 2015). Although still widely used, phototoxicity has limited the use of chemical calcium indicators in exploring intracellular calcium handling of *in vitro* models under prolonged investigation (Shinnawi et al., 2015).

###### Genetically Encoded Calcium Indicators

Genomic engineering has provided novel and innovative tools for the intracellular imaging of calcium ions. The use of ratiometric dyes, such as fura-2, can impair the contractility of cardiomyocytes through unwanted Ca^2+^ chelation and can produce uneven and erroneous dye loading (Robinson et al., 2018). Genetically encoded Ca^2+^ indicators (GECI) offer numerous advantages over small molecule dyes including cell type-specific calcium imaging, homogenous indicator expression and reduced levels of unintentional compartmentalisation (Bassett and Monteith, 2017).

The recombinant gene for the sensor, which is usually a derivative of green fluorescent protein, can be cloned into commonly used laboratory animals or expressed within *in vitro* cell lines following transfection or viral transduction. The precise mechanisms behind the delivery and design of genetically encoded calcium indicators are beyond the scope of this review. Further information can be obtained from Kaestner et al. (2014). Genetically encoded Ca^2+^ sensors are emerging as a promising tool for high-throughput anti-arrhythmic drug development (Wu et al., 2019). However, their use is currently limited by narrow spectral bands and putative disruption of endogenous signalling cascades.

Intracellular calcium imaging using small molecule and genetically encoded indicators have proven insightful in exploring the effects pathogenic variants have on excitation–contraction coupling, arrhythmia and heart failure. When used in conjunction with the optical imaging of voltage-sensitive dyes, it enables a comprehensive assessment of the electrophysiological state of *in vitro* cell models. This is evidenced in Pierre et al. (2021), where both optical action potentials and calcium transients were recorded to assess the impact of a Na_V_1.5 knock-out in iPSC-CM monolayers.

##### Calcium Spark Analysis

Calcium sparks are small areas of localised fluorescence caused by the ephemeral release of Ca^2+^ from the ryanodine receptors of the sarcoplasmic reticulum (Cheng et al., 1993). In contrast to the calcium transient, the calcium spark is a sudden and unsustained release of Ca^2+^ which cannot independently trigger the contraction of the cell. Calcium sparks are the building blocks of the calcium transient and excitation–contraction coupling (Cheng et al., 1996). Highly sensitive calcium indicators that confer a high signal to noise ratio, such as the non-ratiometric dyes fluo-3 and fluo-4, are used to image calcium sparks.

Increases in angiotensin II activity are commonly observed during the development of AF (Goette et al., 2000). The analysis of calcium sparks in atrial cardiomyocytes by Gassanov et al. (2006) helped demonstrate the pro-arrhythmic effects of angiotensin II. Primary atrial cardiomyocytes that were incubated in angiotensin II demonstrated increased frequencies of spontaneous calcium spark production. Such an increase is linked to abnormal cell membrane depolarisation and is thought to contribute to the re-initiation of AF.

##### Compartment-Specific Calcium Imaging

The compartmentalisation of Ca^2+^ sensitive indicators in intracellular organelles was reported as a common problem during early attempts at calcium imaging (Malgaroli et al., 1987). Recently however, indicators have been used specifically to image the flux of Ca^2+^ in organelles including the mitochondria, endoplasmic reticulum and nucleus. Mitochondrial calcium signalling causes the formation of a dynamic buffer which helps control the concentration of cytosolic Ca^2+^ and it is essential for the generation of the ATP required for cardiac contraction (Dedkova and Blatter, 2013; Boyman et al., 2014). Dysfunction of mitochondrial calcium handling can cause oxidative stress and is strongly associated with the development of chronic heart failure and AF (Luo and Anderson, 2013; Xie et al., 2015; Wiersma et al., 2019). Mitochondrial calcium imaging was used effectively by Santulli et al. (2015) to assess the importance of mitochondrial calcium overload in murine post-myocardial infarction heart failure. Cardiomyocytes derived from the mice demonstrated significant increases in cardiac mitochondrial Ca^2+^ and reactive oxygen species levels following myocardial infarction.

Genetically encoded calcium indicators have been particularly useful for calcium imaging in specific organelles, such as the endoplasmic reticulum, Golgi apparatus and mitochondria (Suzuki et al., 2016).

### Computational Cardiac Modelling and Simulations

#### Fundamentals of Computational Cardiac Modelling and Simulation

Computational (*in silico*) cardiac modelling and simulation is a widely used technique to investigate the biophysical processes underlying cardiac pathophysiology, arrhythmias and heart failure at a multiscale level. They provide unique mechanistic insights at high spatio-temporal resolution, to augment experimental and clinical investigations. Detailed experimental characterisation of cardiac electrophysiology mechanisms by techniques, such as voltage clamping, has enabled the generation of mathematical models capable of describing action potential, excitation–contraction coupling and underlying ionic currents of human atrial, ventricular, Purkinje and iPSC-CMs (Courtemanche et al., 1998; Tomek et al., 2019a; Paci et al., 2020; Trovato et al., 2020; freely available https://www.cs.ox.ac.uk/insilicocardiotox/model-repository). Models, such as these, are based upon the pioneering work performed by Hodgkin and Huxley (1952) and Noble (1960) for the neuronal and cardiac action potential, respectively. The models consist of a set of equations characterising the dynamics of transmembrane and sarcoplasmic reticulum ion channels, pumps and transporters.

#### Ventricular and Atrial Cardiac Computational Models

The ToR-ORd model (Tomek et al., 2019a) is the most recent human ventricular cardiomyocyte model and was derived from the O’Hara-Rudy (ORd) model (O’Hara et al., 2011). The ToR-ORd model includes formulations of key current dynamics and can express repolarisation abnormalities promoting the arrhythmic substrate. The models’ parameters can be varied to represent intersubject variability and disease conditions promoting arrhythmogenesis (Dutta et al., 2017; Passini et al., 2017; Zile and Trayanova, 2017; Muszkiewicz et al., 2018). Specifically, simulation studies using human ventricular single-cell models have provided novel insights into the mechanisms behind heart failure associated arrhythmogenicity (Gomez et al., 2014; Mora et al., 2021; Szlovák et al., 2021). Models have also been developed to study the effect of heart failure-associated changes in sub-cellular structures including t-tubules (Hrabcová et al., 2013; Poláková and Sobie, 2013).

Cardiac computational simulations of atrial electrophysiology are commonly performed using models derived from Nygren et al. (1998), Courtemanche et al. (1998) and Grandi et al. (2011). Such models have been used extensively to study the underlying mechanisms behind the most common sustained type of arrhythmia, AF (Grandi et al., 2019). Genetic variation in the two-pore domain acid-sensitive potassium channel TASK-1 (I_TASK_) has been linked to an increased susceptibility of AF and has been shown to cause prolongation of the action potential duration in animal models (Petric et al., 2012; Liang et al., 2014). Schmidt et al. (2015) used a version of the Grandi model to demonstrate that upregulation of I_TASK_ facilitated the pro-arrhythmic shortening of action potential duration *in silico* and that pharmacological inhibition of the channel represented a viable anti-arrhythmic strategy. Tools incorporating single-cell models of different cell types have been developed to predict pro-arrhythmic cardiotoxicity and inform clinical risk stratification of different drugs, specifically anti-arrhythmic drugs (Passini et al., 2017; Sutanto et al., 2019).

#### Applications of Cardiac Computational Modelling and Simulation

Cardiac computational models can be used to comprehensively investigate the mechanisms behind genetic variant associated arrhythmogenicity. Robust models of atrial, ventricular and sinoatrial nodal cellular electrophysiology can be used in conjunction to help researchers reveal the effect that pathogenic variants confer in multiple cardiac cell types. Gain of function variants in the voltage-gated potassium channel gene *KCNQ1* are associated with the development of complex phenotypes including AF and QT prolongation (Hasegawa et al., 2014). Paradoxically, pathogenic variants in *KCNQ1* have also been identified in patients with short QT syndrome 2 (Wu et al., 2015). Zhou et al. (2019) conducted experimentally informed *in silico* simulations using a selection of human atrial, ventricular and sinus nodal models to identify the pathological mechanism behind a gain of function variant of *KCNQ1*. The simulations implicated the elongation of the ventricular action potential duration as a possible cause of conduction delays and QT prolongation.

Integrating biophysical cellular models into anatomical whole-organ and electrical propagation models enables multiscale simulations of cardiac electrophysiology from ionic current to the ECG (Sánchez et al., 2018; Martinez-Navarro et al., 2019; Mincholé et al., 2019). Incorporating experimental mechanistic insights and data on the mechanics of tension development in human cardiomyocytes allows for the construction of human-based electromechanical models capable of representing abnormalities in the ECG and mechanical function caused by disease conditions, such as myocardial infarction (Land et al., 2017; Margara et al., 2021; Wang et al., 2021). They have also been used to investigate mechanical function in a biventricular model under heart failure conditions (Park et al., 2018). Furthermore, three-dimensional *in silico* modelling and simulation has been employed to study arrhythmogenicity of cell therapy using stem cell-derived cardiomyocytes, exploring the effects of graft size, location, anisotropy and ectopic beat propagation (Yu et al., 2019, 2021). Organ level computational studies have furthermore been conducted on the atria, with a specific focus on mechanisms and treatment of AF (Aslanidi et al., 2011; Zhao et al., 2017; Roney et al., 2018). A study by Dux-Santoy et al. (2011) highlighted the relevance of including the cardiac conduction system in whole heart simulations, the absence of which presents a considerable limitation in some three-dimensional studies.

#### Machine Learning

The use of artificial intelligence (AI) and machine learning (ML) presents an exciting opportunity to increase the predictive power of computational models in clinical and experimental arrhythmia research. Definitions of key concepts including deep learning, ML and artificial neural networks as well as examples by which the implementation of AI could change clinical research in cardiac electrophysiology and disease can be drawn from Feeny et al. (2020). In recent years, the generation of clinical data, including cardiac images, ECGs and DNA sequencing status, has occurred at an unprecedented rate. AI methods enable large quantities of complex data to be filtered and analysed to identify causal links that may not be immediately evident.

Supervised machine learning (SML) has been the most widely used form of AI applied to arrhythmia and heart failure research. SML techniques have been employed to categorise iPSC-CM from patients with catecholaminergic polymorphic ventricular tachycardia, long QT syndrome and hypertrophic cardiomyopathy (Juhola et al., 2018). Another study has employed machine learning techniques to classify different phenotypes of hypertrophic cardiomyopathy, the mechanisms behind their heterogeneities and differences in arrhythmic risks (Lyon et al., 2019). These studies highlight the exciting development in applying ML techniques to experimental data and could facilitate significant change in the ways we currently evaluate genetic variants and the increased risk they confer on arrhythmogenesis.

#### Impact and Benefit of Computational Cardiac Modelling and Simulations in Arrhythmia and Heart Failure Research

In summary, computational modelling and simulation has improved our current understanding of cardiac electrophysiology, the development of arrhythmia and the mechanisms underlying heart failure. Experimental and clinical studies are time-consuming, require biological resources and overall can be extremely costly. *In silico* simulation studies provide a cost-effective and complementary technique, which can reduce the amount of necessary *in vitro* and animal models used in the interrogation of cardiac mechanisms. Computational modelling and simulation studies can also precede and drive large scale experimental or clinical studies by predicting a drug’s optimal dose or identifying groups at risk of adverse treatment effects.

*In silico* models and simulations are scalable, detailed and biophysically accurate and can give insights into arrhythmia mechanisms which would be otherwise imperceptible to researchers using experimental data solely. Since computational studies are informed by and based on real data to ensure their clinical relevance, they can sometimes be restricted by the availability of suitable data. Furthermore, computational power is limited, implying that researchers must balance the complexity of their model against its performance. Parallel computing and advances in computer architecture have made advances in addressing these issues (Sachetto Oliveira et al., 2018).

## Discussion

### The Benefit of Combining Research Modalities

**Figure 3:**
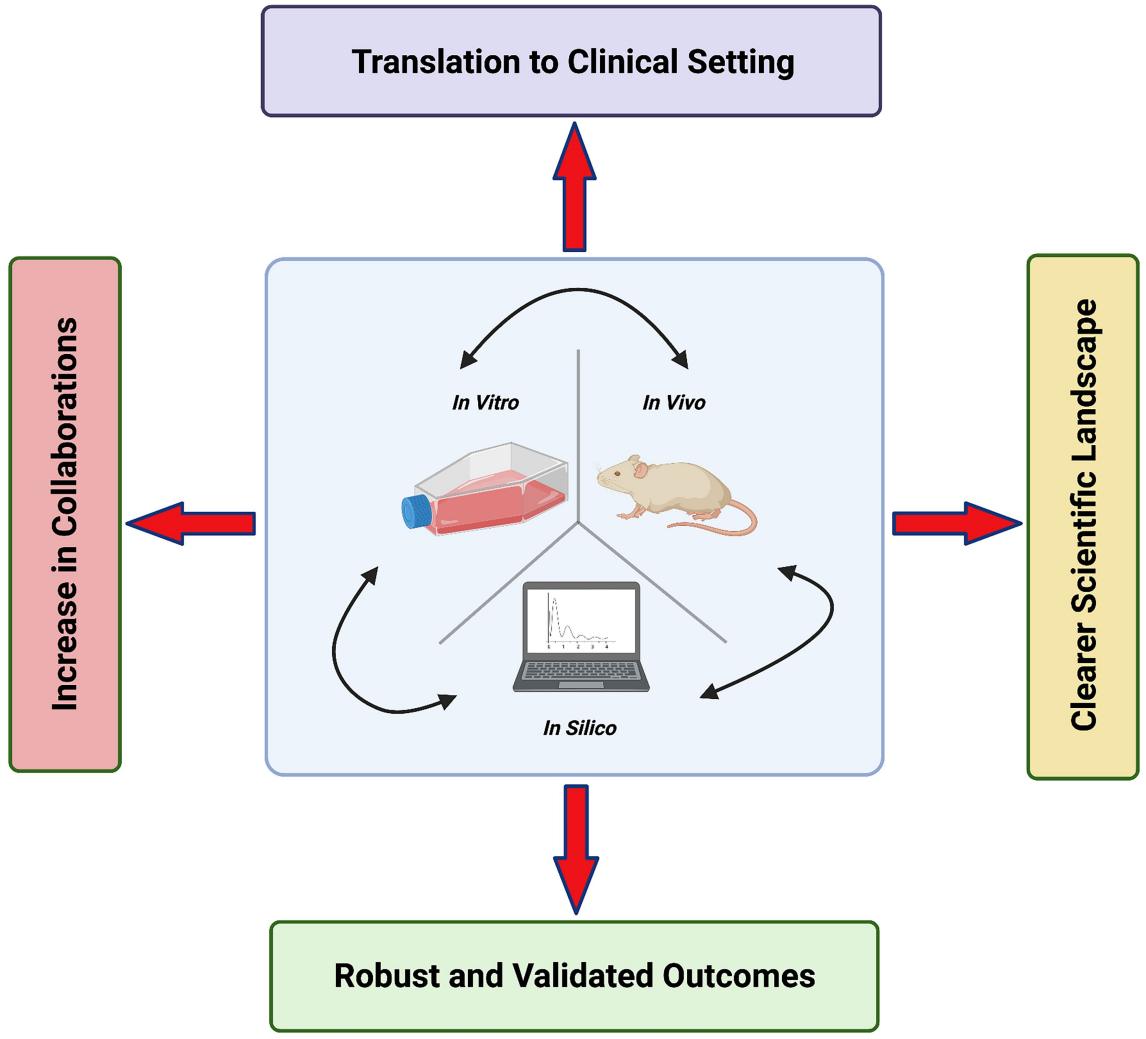
The benefits of a “Team Science” approach in cardiac arrhythmia and heart failure research. Created with Biorender.com.

The relationship between heart failure and arrhythmias is complex and often manifests through diverse aetiologies. Hence, there is benefit in a varied approach to study them, combining the use of *in vitro*, *in vivo* and *in silico* models and using a wide array of experimental techniques. This will overcome the limitations present when using only a single model or a limited toolbox of techniques. However, it requires pulling expertise from various areas and the collaboration of specialists in a “Team Science” approach. The benefits of this approach are outlined in Figure 3. Similarly, Odening et al. (2021) advocate “strategies that combine different methodological approaches” in cardiac electrophysiology research.

*In vivo* and *in vitro* models have been used in conjunction to generate complementary data sets. This is evidenced in Gesmundo et al. (2017), where the group used a number of the models and techniques discussed above to investigate the beneficial effect growth hormone-releasing hormone had on cardiac hypertrophy and heart failure. Elicitation of the drug in immortalised H9c2 cardiac cells (section “Cellular Systems: Immortalised Cardiac Cells”), adult rat ventricular cardiac myocytes (section “Cellular Systems: Primary Cells”) and iPSC-CM (section “Cellular Systems: Induced Pluripotent Stem Cells”) counteracted phenylephrine-induced hypertrophy and reduced expression of hypertrophic genes, such as *Epac1* (Ulucan et al., 2007). *In vivo*, an agonist of the hormone provided complementary results and was able to improve cardiac function and alleviate cardiac hypertrophy in mice with transverse aortic constriction.

The synergistic use of computational modelling and wet-lab experiments is an emerging area with potential to achieve robust, mechanistic and interpretable results. It is exemplified by the combined use of *in vivo* and *in silico* models in Tomek et al. (2019b) where optical mapping data was derived from Langendorff perfused post-myocardial infarction (MI) rat hearts (section “*Ex vivo* Cardiac Preparations”). An increased liability to alternans formation was observed at the border zone when paced at longer cycle lengths. β-Adrenergic receptor stimulation with norepinephrine reduced alternans formation by approximately 60% when elicited in the infarct border zone of retrogradely perfused rat hearts. Results were subsequently reproduced in computer models of the border zone informed on intracellular calcium handling and ion channels. The results obtained in the study, using both *ex vivo* and *in silico* models, supported clinical imaging studies which predict border zone denervation as being pro-arrhythmic (Malhotra et al., 2015). While previous data obtained from animal models have conversely demonstrated sympathetic reinnervation of the border zone post-myocardial infarction as being pro-arrhythmic (Shen and Zipes, 2014). Understanding the effect β-adrenergic receptor stimulation has on the border zone of healed myocardial infarctions (MI) is clinically important, as it can inform treatment. It is routine for patients to be prescribed beta blockers post-MI and for chronic heart failure, as they reduce heart rate and blood pressure and thus decrease myocardial workload (Lange et al., 1983). These examples highlight the relevance of combining different experimental and computational techniques to validate findings and ensure the robustness of predictions for a clinical setting.

### Conclusion

This review has outlined state-of-the-art experimental and computational methods and their relative strengths and weaknesses. The authors conclude that there is not one ideal model or methodology for all studies. Instead, research into arrhythmia and heart failure requires a careful consideration of its goals, resources and scope. Previous studies have shown that a combination of experimental and computational models can provide robust and validated outcomes in a variety of research settings. Such an approach will help to gain detailed mechanistic insights, which are a prerequisite for developing targeted therapies to prevent or at least ameliorate arrhythmias in heart failure patients.

## Author Contributions

All authors listed have made a substantial, direct, and intellectual contribution to the work, and approved it for publication.

## Funding

This work was funded by the National Centre for the Replacement, Refinement and Reduction of Animals in Research (NC3Rs; NC/T001747/1 to KG and MC). Work in KG’s laboratory is funded by the British Heart Foundation (BHF) (PG/19/45/34419 and FS/12/40/29712); the Medical Research Council (MR/V009540/1); and the Wellcome Trust (201543/B/16/Z and 204846/Z/16/Z to UoB). LR is funded by a BBSRC PhD scholarship in collaboration with AstraZeneca (BB/V509395/1). BR is funded by a Wellcome Trust Senior Research Fellowship in Basic Biomedical Sciences (214290/Z/18/Z) and an NC3Rs Infrastructure for Impart Award (NC/P001076/1). AH is funded by the BHF Project grant (PG/17/30/32961) and a BHF Studentship (FS/PhD/20/29093). The Institute of Cardiovascular Sciences, University of Birmingham, has received an Accelerator Award by the British Heart Foundation (AA/18/2/34218). CO’S is funded by a Wellcome Trust (Sir Henry Wellcome Fellowship 221650/Z/20/Z). AR is funded by a BHF Accelerator (AA/18/2/34218). CD is funded by the British Heart Foundation (CRMR/21/290009, PG/21/10545) and the National Centre for the Replacement, Refinement, and Reduction of Animals in Research (35911–259146, NC/K000225/1, NC/S001808/1).

## Conflict of Interest

The authors declare that the research was conducted in the absence of any commercial or financial relationships that could be construed as a potential conflict of interest.

## Publisher’s Note

All claims expressed in this article are solely those of the authors and do not necessarily represent those of their affiliated organizations, or those of the publisher, the editors and the reviewers. Any product that may be evaluated in this article, or claim that may be made by its manufacturer, is not guaranteed or endorsed by the publisher.
